# Dynamics of Bacterial Community Structure in the Rhizosphere and Root Nodule of Soybean: Impacts of Growth Stages and Varieties

**DOI:** 10.3390/ijms22115577

**Published:** 2021-05-25

**Authors:** Soo-In Sohn, Jae-Hyung Ahn, Subramani Pandian, Young-Ju Oh, Eun-Kyoung Shin, Hyeon-Jung Kang, Woo-Suk Cho, Youn-Sung Cho, Kong-Sik Shin

**Affiliations:** 1 Department of Agricultural Biotechnology, National Institute of Agricultural Sciences, Jeonju 54874, Korea; pandiannsp7@gmail.com (S.P.); novis7@korea.kr (E.-K.S.); happykorean@korea.kr (H.-J.K.); phyto@korea.kr (W.-S.C.); younsung@korea.kr (Y.-S.C.); 2 Department of Agricultural Biology, National Institute of Agricultural Sciences, Jeonju 55365, Korea; hyungz@korea.kr; 3 Institute for Future Environmental Ecology Co., Ltd., Jeonju 54883, Korea; 50joo@hanmail.net; 4 Audit and Inspection Office, Rural Development of Administration, Jeonju 54875, Korea; kongsiks@korea.kr

**Keywords:** bacteria, community composition, growth stage, rhizosphere, root nodule, soybean, 16S rRNA gene

## Abstract

Bacterial communities in rhizosphere and root nodules have significant contributions to the growth and productivity of the soybean (*Glycine max* (L.) Merr.). In this report, we analyzed the physiological properties and dynamics of bacterial community structure in rhizosphere and root nodules at different growth stages using BioLog EcoPlate and high-throughput sequencing technology, respectively. The BioLog assay found that the metabolic capability of rhizosphere is in increasing trend in the growth of soybeans as compared to the bulk soil. As a result of the Illumina sequencing analysis, the microbial community structure of rhizosphere and root nodules was found to be influenced by the variety and growth stage of the soybean. At the phylum level, Actinobacteria were the most abundant in rhizosphere at all growth stages, followed by Alphaproteobacteria and Acidobacteria, and the phylum Bacteroidetes showed the greatest change. But, in the root nodules Alphaproteobacteria were dominant. The results of the OTU analysis exhibited the dominance of *Bradyrhizobium* during the entire stage of growth, but the ratio of non-rhizobial bacteria showed an increasing trend as the soybean growth progressed. These findings revealed that bacterial community in the rhizosphere and root nodules changed according to both the variety and growth stages of soybean in the field.

## 1. Introduction

Soybeans (*Glycine max* (L.) Merr.) are one of the world’s most valuable crops, yielding 300 million tons per year [1]. It was first cultivated in China and then spread to other parts of the globe [2]. The global soybean cultivation area is now 128.22 million ha, up 1.9 percent year on year [3]. The use of chemical fertilizers result in environmental perturbations like eutrophication in rivers and lakes as well as global warming. Therefore, we must consider the environmental issues along with the yield for the sustainable agricultural production. Soybeans do not need much fertilizer because they have a symbiotic relationship with rhizobia and arbuscular mycorrhizal fungi, but unwantedly, a lot of fertilizer is used in soybean cultivation. The effective use of rhizosphere microorganisms for the desirable growth of soybeans is necessary for sustainable agricultural production. Biofertilizers containing specific strains of plant growth-promoting rhizobacteria (PGPR) have been used for decades to increase plant nutrient use efficiency and reduce the use of chemical fertilizers and pesticides [4]. Initially, applied biofertilizer interacts with rhizosphere and root-associated microbes and later successfully colonize the roots to benefit the plant [5,6,7,8,9]. Understanding the dynamics and diversity of native microbial communities associated with the roots and rhizosphere is needed to gain insight into such interactions [9,10,11].

Rhizosphere soil is defined as a zone that contains soil that has been affected by roots as well as microorganisms colonizing the root structure [12,13,14]. It acts as an initial filter for subsets of microorganisms that colonize the root as endophytes [15]. Plants can actively modulate the assembly of beneficial microbiome [6,16,17]. Microorganisms inhabiting the rhizosphere play an important role in plant health and defense [18,19,20], stress response [21], nutrition [22,23], and plant growth promotion [24]. The plant root exudates mediate interactions between microbial communities present in the roots and rhizosphere [25,26,27]. Plant roots release 5–21% of photosynthetically fixed carbon in the forms of soluble sugars, amino acids, and secondary metabolites, which are used by the rhizosphere microbial communities [26,27,28,29]. The community structure of rhizosphere is affected by the source and amount of substances released from the roots [14], root architecture [30], species or genotype of plants [23,31,32], and growth stages [33].

Since soybean is an important nutritional and economic crop it is imperative to study the dynamics of rhizosphere community composition with high-resolution profiling methods. High-throughput sequencing technology enabled us to study plant-associated microbial communities at high resolution. Studies on rhizosphere microorganisms associated with plant roots have been conducted in *Arabidopsis thaliana*, *Hordeum vulgare* [15,16,34,35], corns, and soybeans [22,36]. Despite these fundamental studies, the truth is that there is a scarcity of research on the roots and rhizosphere microorganisms in commercial and non-model plants. Since soybeans have a symbiotic relationship with nitrogen-fixing (N_2_) rhizobia and enhances its N_2_-fixing capability, a number of studies have been conducted to better understand the processes and signaling pathways [24,37,38,39]. Based on the previous studies, the effective interaction between soybeans and beneficial root nodule bacteria improves plant health, yield, and resilience [24,39]. The beneficial effects of *Bradyrhizobium* spp. on the soybean nodule formation and N_2_ fixation are well known and members of the *Bradyrhizobium* genus have been confirmed to influence soybean growth and yield [40,41]. However, little is known about the variation in the residential bacterial population in root nodules based on the growth stage. As the first step in elite soybean breeding, understanding the diversity of the root nodule microbiome is crucial. The frequency and ecotypes of atypical bacterial taxa in root nodules of soybean and other legume plants have been identified using culture-dependent sampling methods, and microbiome sequencing [42,43,44]. Despite their findings, more concerns remain such as the influence of growth stages, cultivars, and their mechanisms in the dynamics of microbial community composition.

To fill these lacunae, we examined the interactions between soybeans and microorganisms, soybeans and root nodule bacteria, and rhizosphere and root nodule bacteria in the field environment. Therefore, population-level BioLog substrate utilization assay was used to examine physiological properties of microbial species, and 16S rRNA gene sequencing was used to assess the dynamics of community composition between rhizosphere and root nodule bacteria by the variety and growth stages of soybean.

## 2. Materials and Methods

### 2.1. Plant Materials

*Glycine max* (L.) Merr. cv. Kwangan (KA), Poongsannamul (PS), Poongwon (PW), and Taekwang (TK) used in this study were obtained from the National Institute of Crop Science, Jeonju, South Korea.

### 2.2. Field Construction and Soil Sample Preparation

**Figure 1:**
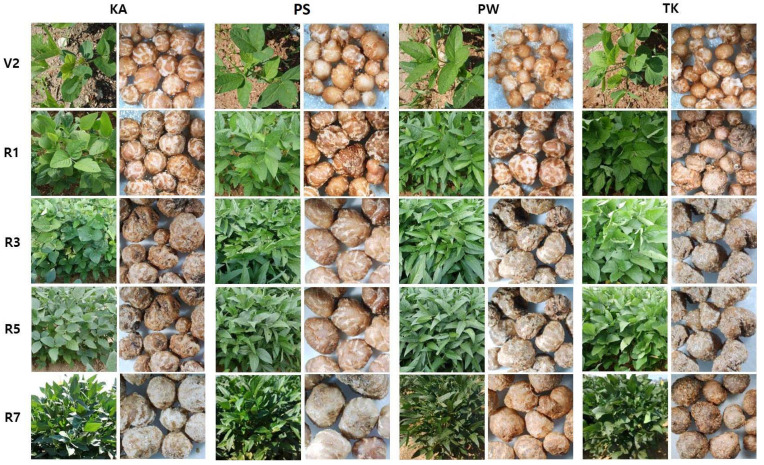
Morphology of four varieties of plants and root nodules based on the various growth stages (V2. R1, R3, R5, R7) of soybean. KA, Kwangan; PS, Poongsannamul; PW, Poongwon; TK, Taekwang.

The experimental plot for assessing the bacterial community structure in four soybean varieties (KA, PS, PW, and TK), was established in the field of the National Institute of Agricultural Sciences, Rural Development Administration, Jeonju, Korea. For each soybean variety, three replicates of the experimental plot were made. Each experimental plot was 4 × 4 m² in size. Three replicates of soil samples were collected from the soils prior to planting (bulk soil), as well as soil samples from the rhizosphere and root nodules of each stage, including vegetative (V2) and reproductive stages (R1, R3, R5, and R7). Morphology of plants and root nodules in different varieties and growth stages was shown in Figure 1.

To obtain rhizosphere soil, we collected soybean with its root, completely removed bulk soil and then collected soil that is attached to the root as much as possible using sterile brushes. Rhizosphere soil samples were immediately transported to the laboratory in a cool container (4 °C) within 2 h. Immediately after the homogenization and passing through a 2 mm sieve, 1.5 g of each sample was used in the BioLog substrate utilization assay to avoid any changes in microbial communities while storing soils. The residual soil was preserved at −30 °C until subsequent DNA extraction. 

### 2.3. Root Nodule Preparation

Soybean root nodules were removed from roots, washed, and dried overnight at 65 °C (oven-dried), then stored at room temperature in vials containing silica gel until experiment time [45]. Initially, the root nodule was rehydrated in sterile water until completely swollen, then treated with 95% ethanol for 5 min, sterilized with 3% sodium hypochlorite solution for 3 min, and washed 5 times with sterile distilled water for DNA extraction. To obtain rhizobial cell suspension, each root nodule was loaded into a 5 mL micro centrifuge tube, 0.1 mL of TE buffer was added, and mechanically pressed using a sterile plastic pipette tip. The cell suspension was transferred to a new tube and the cells were collected by a simple centrifugation process at 15,000 rpm for 5 min at 4 °C. The supernatant was removed, and DNA was extracted using rhizobial cells.

### 2.4. Community-Level Physiological Profiling (CLPP) Analysis

The potential of the microbial community functions was investigated through CLPP analysis by inoculating soil samples into each well of the EcoPlate (Biolog, Hayward, CA, USA). For this experiment, soil samples were dissolved in sterile water at a 1:10 (*w/w*) ratio and stirred for 10 min at 200 rpm. Then, 150 μL of the supernatant was taken to fill each EcoPlate wells. After that, it was incubated at 20 °C, and the color change in each well was measured and analyzed using Multiskan Ascent at 595 nm wavelength in every 2 h for 24 h (Thermo Labsystems, Finland). Each EcoPlate was divided into three identical zones, serving as replicates, and the absorbance value of each carbon source was corrected by subtracting the absorbance value of the control well (without any carbon substrate); negative values were set to zero. The mean of the 31 corrected values was used to measure Average Well Color Development (AWCD) for each replication and sampling time [46,47]. 

### 2.5. PCR Amplification and Illumina Sequencing

Metagenomic DNA was extracted from bulk soils, rhizosphere soils, and root nodules of soybeans using the Fast DNA Spin Kit (Qbiogen, Carlsbad, CA, USA) according to the manufacturer’s instructions. PCR amplification was carried out with extracted DNA using universal eubacterial primers targeting the V3-V4 hypervariable regions of the 16S rRNA gene. For bacterial amplification, primers of 341F (5′-TCGTCGGCAGCGTC-AGATGTGTATAAGAGACAG-CCTACGGGNGGCWGCAG-3′; underlining sequence indicates the target region primer) and 805R (5′-GTCTCGTGGGCTCGG-AGATGTGTATAAGAGACAG-GACTACHVGGGTATCTAATCC-3′). The amplifications were carried out under the following conditions: initial denaturation at 95 °C for 3 min, followed by 25 cycles of denaturation at 95 °C for 30 s, primer annealing at 55 °C for 30 s, and extension at 72 °C for 30 s, with a final extension at 72 °C for 5 min. Then, secondary amplification for attaching the Illumina NexTera barcode was performed with i5 forward primer (5′-AATGATACGGCGACCACCGAGATCTACAC-XXXXXXXX -TCGTCGGCAGCGTC-3′; X indicates the barcode region) and i7 reverse primer (5′-CAAGCAGAAGACGGCATACGAGAT-XXXXXXXX-TCTCGTGGGCTCGG-3′). The condition of secondary amplification is equal to former one except the amplification cycle set to 8.

The PCR product was confirmed by using agarose gel (1%) electrophoresis and visualized under a Gel Doc system (BioRad, Hercules, CA, USA). The amplified products were purified with the CleanPCR (CleanNA, The Netherlands). Equal concentrations of purified products were pooled together and removed short fragments (non-target products) with CleanPCR (CleanNA, The Netherlands). The quality and product size were assessed on a Bioanalyzer 2100 (Agilent, Palo Alto, CA, USA) using a DNA 7500 chip. Mixed amplicons were pooled and the sequencing was performed using an Illumina MiSeq Sequencing platform (Illumina, San Diego, CA, USA) at Chunlab, Inc. in Seoul, South Korea, according to the manufacturer’s instructions.

### 2.6. Bioinformatics Analyses

Processing raw reads starts with quality check and filtering of low quality (<Q25) reads by Trimmomatic 0.32. After QC pass, paired end sequence data are merged together using PANDAs eq. Primers are then trimmed with ChunLab’s in house program at a similarity cutoff of 0.8. Nonspecific amplicons that do not encode 16S rRNA are detected by HMMER’s hmmsearch program with 16S rRNA gene profiles. Sequences are de-noised using DUDE Seq and non-redundant reads are extracted by UCLUST clustering. The EzBio Cloud database is used for taxonomic assignment using USEARCH (8.1.1861_i86linux32) followed by more precise pairwise alignment UCHIME and then on chimeric 16S rRNA gene database from EzBioCloud are used to detect chimera on reads that contain a less than 97% best hit similarity rate. Sequence data are then clustered using CD-HIT and UCLUST. The alpha diversity indices and rarefaction curves are estimated by in-house code. 

### 2.7. Statistical Analysis

Results are presented with the mean ± standard deviations of triplicate experiments. One-way analysis of variance (ANOVA) with Tukey’s HDS (honestly significant difference) test (*p* ≤ 0.05) was performed with GraphPad Prism ver. 5.0 (GraphPad Software, Inc., San Diego, CA, USA).

## 3. Results

### 3.1. BioLog Substrate Utilization Assay

**Figure 2:**
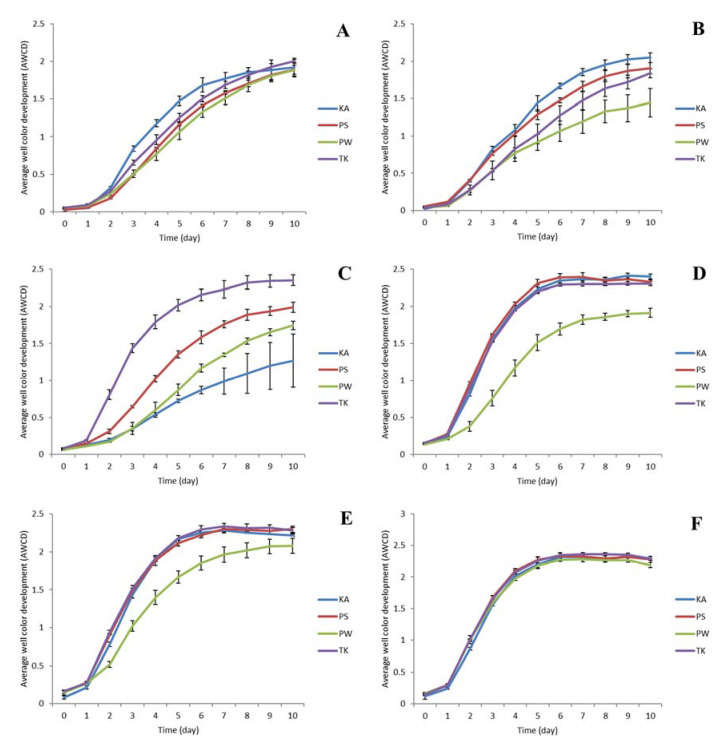
Changes of AWCD according to incubation time of rhizosphere soil microbes based on the growth stages (V2, R1, R3, R5, R7) of soybean. Values indicate mean (*n* = 3) ± standard deviation. (**A**), Soil before planting soybean; (**B**), V2 stage; (**C**), R1 stage; (**D**), R3 stage; (**E**), R5 stage; (**F**), R7 stage.

The BioLog substrate utilization assay was carried out with bulk soil and rhizosphere soil of various growth stages (V2, R1, R3, R5, and R7) of all the four soybean varieties. The color intensity was determined by calculating the average well color development (AWCD). When the same amount of soil was used, the AWCD of rhizosphere soil was 1.5–3.0 times higher than that of bulk soil throughout the growth stages of soybean (Figure 2A–F). Similarly, differences in the varieties of soybean also found to be influenced in the values of AWCD (Figure 2A–F). These results indicating that the metabolic capabilities of the rhizosphere soil were higher than those of the bulk soil and the physiological profiles of the two classes of soil were very different (Figure 2A–F; Table S1)

### 3.2. Alpha Diversity Analysis

A total of 721,643 sequences was obtained from 12 soil samples (soil where 4 varieties of KA, PS, PW, and TK are to be planted, 3 repetitions) of the bulk soil. A total of 3,433,735 sequences was obtained from 60 soil samples (4 varieties same as the bulk soil, 3 repetitions, 5 stages of soybean growth: V2, R1, R3, R5, and R7) of the rhizosphere soil. A total of 2,617,554 sequences was obtained from 60 root nodule extracted DNA samples (4 varieties, 3 repetitions, and 5 stages of soybean growth: V2, R1, R3, R5, and R7). These reads were clustered into 13,628 OTUs (Table S2). The alpha diversity of rhizosphere soil and the root nodule bacteria was affected by the variety and growth stage of the soybean (Table 1 and Table 2; Figure S1A–F and  Figure S2A–F). In the rhizosphere, the Sobs, Chao1, and ACE, which denote species richness (number of OTUs), that increased in the V2 and R1 stages as compared with the bulk soil where the soybean was not planted. Subsequently, these decreased from the R3 to R7 stages (Figure S1A–C). The Shannon, InvSimpson, and NPShannon, which refer to the species diversity index, showed a similar trend and increased until the V2 or R1 stage and decreased from the R3 to R7 stages (Figure S1D–F). Contrarily, the alpha diversity of the root nodule bacteria showed a different trend from the bulk and rhizosphere soil (Table 2; Figure S2A–C). The Shannon, InvSimpson, and NPShannon all seemed to increase from the V2 to R7 levels, similar to the Sobs, Chao1, and ACE (Table 2; Figure S2A–F).

### 3.3. Phylogenetic Structure Analysis

**Figure 3:**
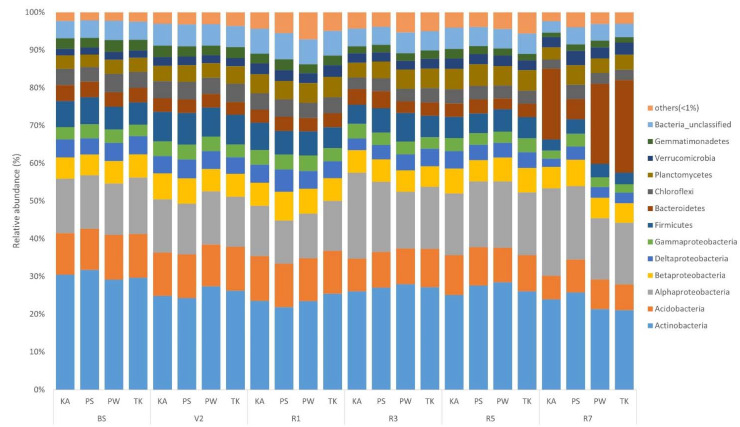
Relative abundance of major phyla in soil before soybean is planted and rhizosphere soil based on the variety and growth stages (V2, R1, R3, R5, R7) of soybean. KA, Kwangan; PS, Poongsannamul; PW, Poongwon; TK, Taekwang. Each bar represents the average relative abundance of triplicates.

**Figure 4:**
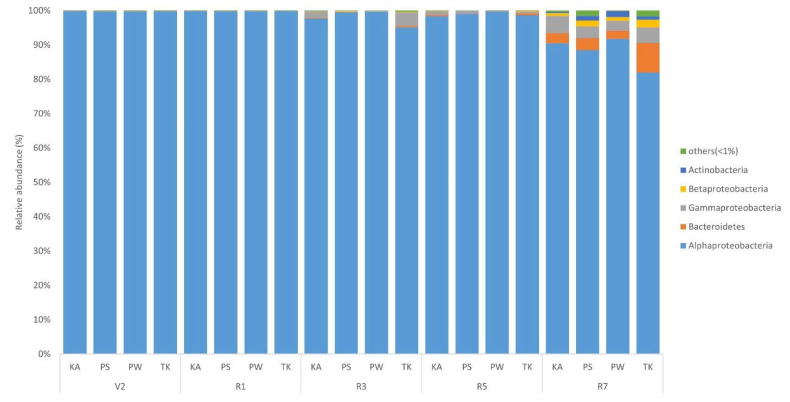
Relative abundance of major phyla presents in root nodule based on the variety and growth stages (V2, R1, R3, R5, R7) of soybean. KA, Kwangan; PS, Poongsannamul; PW, Poongwon; TK, Taekwang. Each bar represents the average relative abundance of triplicates.

**Figure 5:**
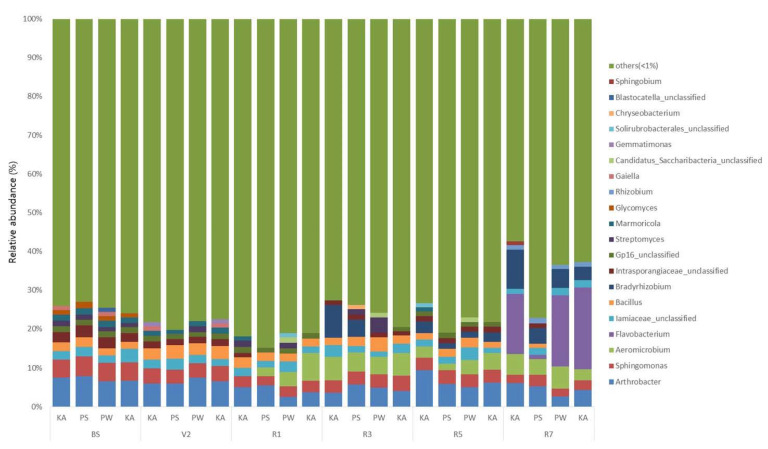
Relative abundance of genus in rhizosphere based on the variety and growth stages (V2, R1, R3, R5, R7) of soybean. KA, Kwangan; PS, Poongsannamul; PW, Poongwon; TK, Taekwang. Each bar represents the average relative abundance of triplicates.

**Figure 6:**
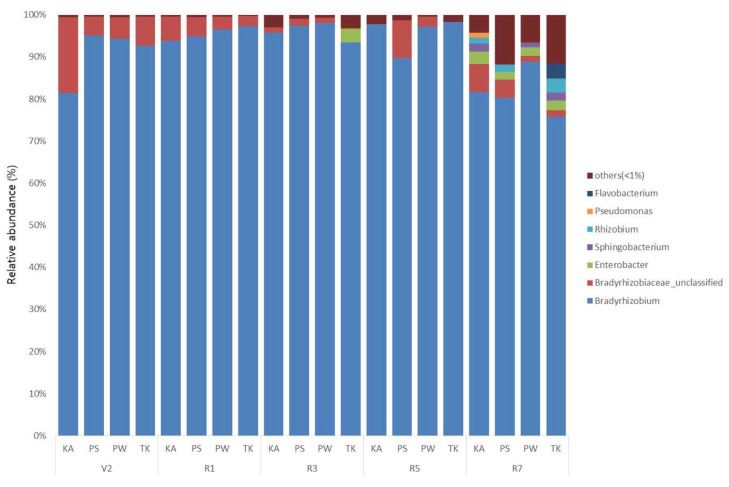
Relative abundance of genus in root nodule based on the growth stages (V2, R1, R3, R5, R7) of soybean. A, B. The bacterial community structure in the bulk soil was different from other growth stages and the stages V2, R1, R3, and R5 of the rhizosphere soil were clustered together; however, the R7 stage is also different from those of other growth stages. These results indicate that after planting the bacterial communities in the soil differed compared to those of bulk soil. Moreover, among the different growth stages the bacterial community composition at the R7 has been differed drastically.

**Figure 7:**
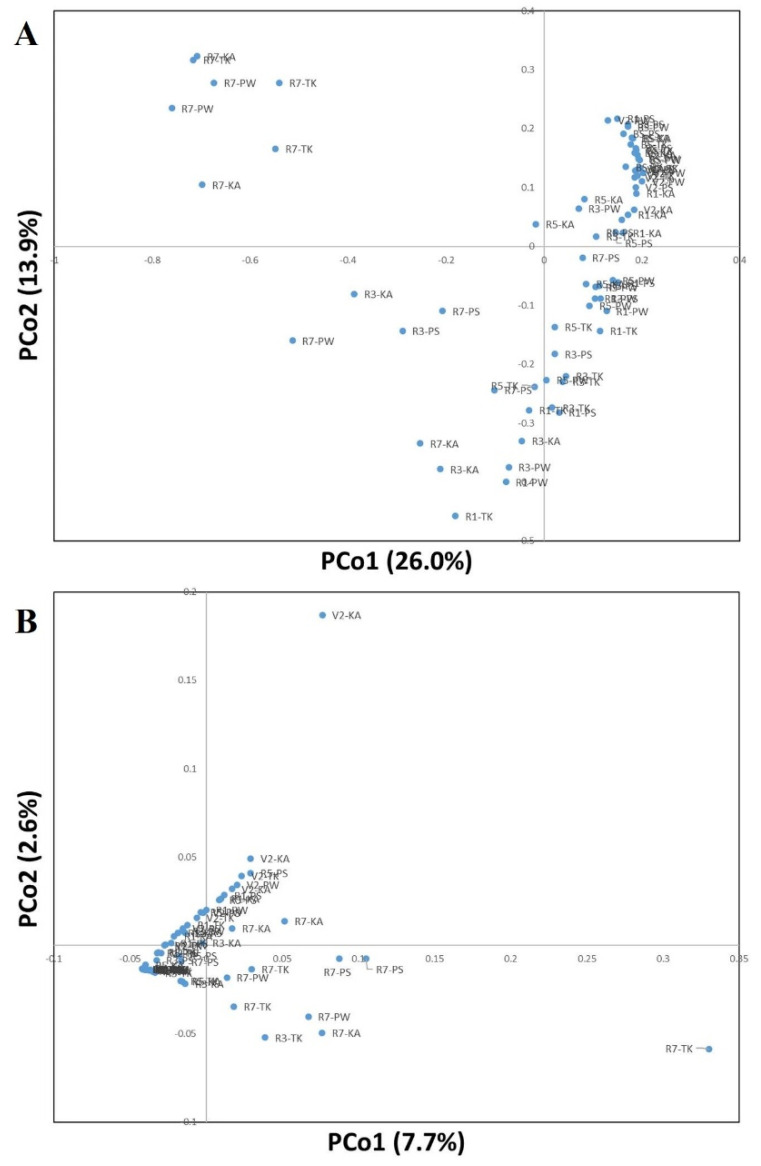
Principal component analysis (PCA) plotted for all the growth stages in the four soybean varieties. PCA results showing the distribution and variation of bacterial communities in the (**A**), Rhizosphere; (**B**), Root nodule.

The sequences of rhizosphere and root nodule bacteria were classified into 36 phyla. Throughout the growth stages, *Actinobacteria* was the most abundant phyla in the bulk soil and rhizosphere, followed by Alphaproteobacteria, Acidobacteria, and Betaproteobacteria (Figure 3; Figure S3A–D). As the growth stage progressed from the V2 stage, Actinobacteria displayed a tendency to decrease (Figure S3C). The distribution ratio of Alphaproteobacteria increased after the R3 stage while, Acidobacteria decreased after the R3 stage (Figure S3B,C). Bacteroidetes increased the most during the R7 stage except for PS (Figure S3E). Chloroflexi, Firmicutes, Gemmatimonadetes, Deltaproteobacteria, and Gammaproteobacteria showed a tendency to decrease as the growth stage progressed (Figure S3F–J). In root nodule, Alphaproteobacteria showed the highest distribution ratio in the overall soybean growth stage at the phylum level (Figure 4; Figure S4A). Bacteroidetes, Gammaproteobacteria, Betaproteobacteria, Actinobacteria, and Verrucomicrobia showed a tendency to gradually increase as the growth stage progressed (Figure S4B–F). At the genus level, on average, *Arthrobacter* was the most predominant in bulk soil and rhizosphere, followed by *Sphingomonas* (Figure 5; Figure S5A,B; Table S2). Although there is a difference in the distribution ratio according to soybean varieties, *Aeromicrobium* increased in the R1 stage (except for KA), and *Flavobacterium* increased significantly in R7 stage (Figure S5C,D). *Bradyrhizobium* was also different for each cultivar but showed a tendency to increase from the R3 stage (excluding PW and TK) (Figure S5E). At the genus level, *Bradyrhizobium* (75.8–98.4%) was dominant in root nodules (Figure 6; Figure S6A; Table S2). Looking at the distribution of microorganisms by growth stage except for *Bradyrhizobium*, the distribution of *Bradyrhizobiaceae*_unclassified was dominant at the V2 and R1 stages and the same for the R3 stage (Figure 6). Unlike the previous stages, the distribution of *Rhizobium*, *Streptomyces*, *Enterobacter*, *Spingobacterium*, *Flavobacterium*, and *Achromobacter* increased from the R3 stage to the R7 stage (Figure S6B–G). Principal component analysis (PCA) results showing the distribution and variation of bacterial communities in the rhizosphere and root nodule of all the growth stages in the four soybean varieties (Figure 7).

## 4. Discussion

Rhizosphere bacterial communities which are important for the growth and yield of crops are often influenced by the plants [6,48]. Since soybean is an important crop, there are few studies on the changes in bacterial communities in the rhizosphere [24]. However, there is no report that comparatively analyzed the bacterial community variation in the rhizosphere and root nodules based on the growth stages of soybean. This is the first study compared the dynamics of bacterial community structures in the rhizosphere and root nodule of four varieties at different growth stages to gain a better understanding of bacterial interactions with soybean. Interestingly, we found that bacterial communities in the rhizosphere soil and root nodules varied in different growth stage and the degree of change differ by varieties also. It is obscure that the timing of significant changes, for instance, from which stage of the growth does the bacterial community changes and whether these changes occur organically in the rhizosphere and root nodule of soybean. Such changes will inevitably affect the seed yield and N_2_ fixation efficiency, and understanding these changes is critical for the soybean breeding of development of high-yield varieties.

A BioLog assay was used to obtain community-level substrate utilization profiles. According to the results, it is concluded that the soybean cultivar and growth stage had an effect on the average well color production (AWCD) of each plate. Except V2 stage, the AWCD of the rhizosphere soil was higher than that of the bulk soil where soybeans were not planted. This is consistent with the findings of Sugiyama et al., [24], who found that the AWCD of the rhizosphere soil is 1.5-3 times that of the bulk soil. Moreover, the activity of soil enzymes tends to increase as the plant grows. In terms of the cultivar, the soil enzyme activity of PW soybean was lower than that of other soybeans. The reason for the variations in soybean cultivar and growth stage should be investigated further. It is expected that the difference in root exudates passing through the root, as a function of cultivar and rearing period, is supposed to result in a difference in rhizosphere soil enzyme activities [24]. However, at the R7 level, there was no difference in soil enzyme activity between cultivars.

The 16S rRNA gene sequencing results depict that both the growth stage and variety had an impact on the dynamics of the bacterial community in the rhizosphere and root nodule. The alpha diversity of the four soybean cultivar at varying growth stages showed conflicting results. More concretely, the Sobs, Chao1, and ACE, along with Shannon, InvSimpson, and NPShannon, increased until the V2 or the R1 stage and then decreased from the R3 to R7 stages (lower than the bulk soil) in the bulk and rhizosphere soil. This suggests that compared with the bulk soil, bacterial richness, and evenness increased in the V2 and R1 stages, and then decreased from the R3 to R7 stages after soybean was planted. It was hypothesized that the diversity of microbes in the rhizosphere soil increases in the early stage of cultivation, but the diversity decreases in the late stage of growth through the peak growth period, and newly dominated bacteria become to reside in rhizosphere. In terms of the alpha diversity at the root nodule, unlike that in the bulk soil and the rhizosphere soil, the species richness index increased over the growth stage. The species diversity index did not exhibit a significant difference from the V2 to R5 stages, but rapidly increased at the R7 stage. This is due to the fact that the number of specific microorganisms involved in plant growth (in particular N_2_ fixation bacteria) increased at the early stages of growth [49]. However, as the number of bacteria increased significantly in the late growth stage, the species evenness also increased, which is possibly due to the presence of various genera with different functional activities.

In rhizosphere soil, *Actinobacteria* was most abundantly distributed phylum in bulk and all over the growth stages, followed by Alphaproteobacteria and Acidobacteria. This is consistent with reports of a high abundance of Proteobacteria, Actinobacteria, Bacteroidetes, and Acidobacteria in legumes such as soybean and alfalfa [42,50]. The phylum that showed the greatest change was Bacteroidetes, which showed a significant increase at the R7 stage compared to the V2 stage when soybean root nodules begin to form in soybean varieties except PS cultivar. The distribution ratio of Bacteroidetes differs by soybean variety as well as the growth stage. The phylum Bacteroidetes was classified under the secretion system type IV functional traits which is possibly involved in the symbiotic interactions between bacterial and other organisms in the soybean rhizosphere [22].

OTU-based PCA analyses shown that there are changes in the rhizosphere bacterial communities by stage of soybean growth, which suggests that the formation of a unique bacterial community in the rhizosphere soil by growth stage. In this study, the genera *Arthrobacter* and *Sphingomonas* were the most abundant in the rhizosphere. It is reported that *Arthrobacter* sp. are capable of fixing N_2_ and solubilizes phosphate to promote the growth of wheat plants [51] and *Sphingomonas* is known to produce indole-3-acetic acid [52]. *Aerobacterium* had a higher distribution ratio in soils at the V2, R1, R3, R5, and R7 stages than bulk soil, and showed an increasing trend with the progress of the growth stage. *Aeromicrobium* is a member of the Actinobacteria family that is present in the soil at a high concentration. Actinobacteria play an important role in resisting disease and promoting growth of plants [53]. Miller et al. [54] reported first that the genus *Aeromicrobium* produces macrolide antibiotic erythromycin, and based on these results, it was assumed to play an important role in disease suppression. *Bradyrhizobium* increased as the soybean rearing stage progressed, and its distribution ratio was the highest at the R7 stage. Soybeans have a symbiotic relationship with rhizobia, such as *Bradyrhizobium japonicum* and *Bradyrhizobium elkanii*, and receive 50–60% N_2_ through the atmospheric N_2_ fixation at the root nodule. The maximum N_2_ fixation occurs between the R3 and R5 stages of soybean growth and decreases between the R5 and R7, which are the seed-filling stages [24]. Since, the N_2_ fixation decreases at the R7 stage, the necessity of *Bradyrhizobium*, which functions for N_2_ fixation, at the root nodule is found to decrease compared to the R3 or R5 stages. In this study, the ratio of *Bradyrhizobium* for the root nodule decreased in the R7 stage, but in the rhizosphere it is found to be increased. Similarly, the increase in *Bradyrhizobium* in the rhizosphere soil from the V2 to R5 can be attributed to the chemoattractant reaction of the soybean root exudate for N_2_ fixation.

The distribution ratio of *Rhizobium* increased at the R7 stage compared to the BS, V2, R1, R3, and R5 stages. Various *Rhizobia* colonize soybean roots, form root nodules, and provide important benefits to plants through N_2_ fixation [55]. The genus *Rhizobium* was also found in wheat and rapeseed roots and is known to account for 2% of the total bacterial population [56]. Although endophytic rhizobia have been reported in wheat and rapeseed, there was no conclusive evidence that they contribute to symbiotic N_2_ fixation. Sharma et al. [57] discovered *Rhizobium* in the root interior of wheat and reported that it increases seedling shoot and root length by producing indole-3-acetic acid. The genus that showed greater change based on the growth stage was *Flavobacterium*, which showed little change at the bulk soil, V2, R1, R3, and R5 stages but increased significantly at the R7 stage. *Flavobacterium* has been reported as a PGPR [58]. Although more research is needed on why *Flavobacterium* increased rapidly at the R7 stage, *Flavobacterium* was found to be involved in auxin production and P-solubilization. Furthermore, the needs of verifying the effect of *Flavobacterium* on plant growth and yield needs were reported [58].

In root nodule, the phylum Alphaproteobacteria was exclusively dominant, followed by Bacteroidetes, Gammaproteobacteria, Betaproteobacteria, Actinobacteria, Verrucomicrobia, Firmicutes, and Deltaproteobacteria. In the results of the OTU analysis, *Bradyrhizobium* was dominant in root nodules all over the growth stages and varieties. In addition, several subdominants (1–2%) and a wide variety of rare OTUs were distributed, suggesting the plant–bacterial mutualistic reactions in a unique way. Questions arise about the high diversity of microorganisms in these root nodules and the function of rare OTUs. Furthermore, how such diversity of root nodule microbes can benefit plant resilience and ecosystem stability as the growth stage progresses. Sharaf et al., [59] stated that the functional importance of non-rhizobial bacteria is unknown, but for soybeans, non-rhizobia bacteria would have a significant impact on energetics, symbiosis, and nutrient exchange. The positive effect of *Bradyrhizobium* sp. on soybean nodulation and of bacterial-fixed N_2_ on plant tissue incorporation are well-known [60]. It is also known that different *Bradyrhizobium* species and strains have different effects on growth and yield of the soybean. However, little is known about natural variation in root nodule bacterial communities based on the different growth stages. The symbiotic relationship between N_2_-fixing bacteria and soybean have developed root nodules as an only ecological niche for N_2_ fixation. However, concerns exist about the diversity of N_2_-fixing bacteria in root nodules, as well as whether they can share space with bacteria from other genera or families. Although culture-dependent sampling and microbiome sequencing have described the occurrence of atypical bacterial taxa, biovars, and strains present in root nodule of legumes other than soybean. However, less is understood about how these atypical bacterial communities vary by soybean variety and their functional roles [1].

In this study, there were *Bradyrhizobium* sp. and other various non-*Bradyrhizobium* bacteria in the root nodules, which showed a variation according to the soybean variety as well as the stage of soybean growth. In the early stages of growth, *Bradyrhizobium* sp. was dominant in the root nodules; however, the ratio of non-*Bradyrhizobium* gradually increased towards the R7 stage, based on the variety. The ratio that non-*Bradyrhizobium* was accounted for the most in the root nodules was 24.2% when targeting the rhizosphere soil of one variety (TK) at the R7 stage. According to Sharaf et al. [59], atypical *Bradyrhizobium* sp. reached ~40%, and such diversity comes from isolating pooled plants that incorporated nodules without subdividing the growth stage of soybean varieties. A taxon with the highest distribution ratio in the root nodules was Alphaproteobacteria, and a genus with the most change was *Rhizobium* excluding *Bradyrhizobium*. Rhizobia colonize soybeans and form root nodules, which offer essential benefits of plants by fixing nitrogen [61].

A phylum with the greatest change of the distribution ratio in the root nodules following Alphaproteobacteria was Bacteroidetes and Gammaproteobacteria, and the genera that showed great change were *Flavobacterium* (Bacteroidetes) and *Pseudomonas* (Gammaproteobacteria). The *Flavobacterium* sp. IR29-16 and other strains have been reported to contribute significantly to the growth of plant roots in length through IAA production [62]. *Pseudomonas* was known to produce siderophore. Siderophore does not appear to have a direct effect on promoting plant growth, but the majority of seed endophytes have this trait, indicating that they play an important role in plant development. Fluorescent *Pseudomonas* can colonize a variety of ecological niches, which is reflected in their iron uptake system with a high degree of diversity [63]. Endophytes that produce siderophores like *Pseudomonas* strain have a selective advantage over other bacteria or pathogens by stealing their irons [64,65]. Gammaproteobacteria are a phylum that also showed a big difference in root nodules by stage of soybean growth. *Enterobacter*, which also belongs to Gammaproteobacteria, is an OTU whose relative frequency is increased in root nodules than rhizosphere. Free-living *Enterobacter* has been reported to be capable of nitrogen fixation, and certain species, such as *E. cloacae*, have been involved in nitrogen fixation in plants and isolated from the rhizospheres of wheat, sorghum, and rice. Enterobacteria in rhizosphere are known to solubilize organic phosphorus with phosphate and support plant availability [66,67]. *Serratia* sp., which also belongs to Gammaproteobacteria, is an OTU whose relative frequency is increased in root nodules than rhizosphere. *Serratia* sp. have been reported to enhance soybean nodulation by *Bradyrhizobium*, which is the evidence that *Serratia* promotes *Bradyrhizobium* growth by producing Fe-chelates [68,69,70].

Other than Gammaproteobacteria, phylum Betaproteobacteria was found with the greatest change in the root nodules based on the growth stage. The genus showing the greatest variations in the phyla Betaproteobacteria was *Achromobacter.* The *Achromobacter* spp. have been reported to exist in soils [13] and plants [71]. Although they are associated with several clinical diseases as human pathogens, many *Achromobacter* strains have been reported to have PGP properties in the literatures [71,72]. For example, *Achromobacter* sp. strain EMCC1936 can produce IAA, gibberellin, and solubilizing rock phosphate and increase vegetative growth and yield parameter of tomatoes based on in vitro analyses and a greenhouse experiment [73]. Genus *Variovorax* showed an increased frequency in the root nodules at the R7 stage. Similarly, *Variovorax* has been reported to appear at a high frequency in the rhizosphere and root of canola [74,75]. *Mycobacterium*, a rare bacterium that belongs to Actinobacteria, is also found in soybean root nodules. This genus is known to have numerous interactions with other taxa, suggesting that rare but essential species can play an important role through dense connection with other groups [22]. Consistently and highly linked bacterial taxa with other groups play a potentially important role in community structures and ecological functions [76]. The root nodules were initially dominated by bacteria that play a key role in nitrogen fixation and gradually shifted to microbes that were functionally diverse on the late stage of growth. *Streptomyces*, a genus that belongs to the same Actinobacteria and is distributed in root nodules, is known to reduce infection of pine roots by *Fusarium* sp. and *Armillaria* sp. As for *Microbacterium* that belongs to the same Actinobacteria, *Microbacterium* sp. P27 is known to have indole-3-acetic acid production, ammonia production, and 1-aminocyclopropane-a-carboxylate deaminase activity [77]. Verrucomicrobia is a phylum that showed the next greatest change, and *Verrucomicrobium* showed the greatest change among the genera that belong to this phylum. The reason for a high abundance at the R7 stage in the root nodules remains unknown, but an oligotroph like Verrucomicrobia and Acidobacteria is known to be more abundant in soils with poor nutrients [78]. *Paenibacilli* that belong to Firmicutes were abundant in the root nodules at the R7 stage and are known to reduce water-deficit stress in a legume by reducing stress-signaling hormones [79]. 

## 5. Conclusions

In conclusion, the dynamics of bacterial community structure in soybean rhizosphere and root nodules exhibited great variations based on their variety and growth stage, which both high-throughput sequencing technology and the BioLog EcoPlate assay confirmed. According to physiological activities assessed by the BioLog assay, the metabolic capabilities of rhizosphere soil were higher than bulk soil during the growth stages. Further, the dynamics of bacterial communities were varied between bulk soil and rhizosphere based on the growth stages and varieties of soybean. Especially, it has few PGPR genera which are highly abundant and specific to the growth stages. The root nodule specific bacterial communities also found to be differed according to the growth stages and the variety. This study sheds light on the effective bacterial community structure variation on the field which could be useful for the future utilization of microbial resources in leguminous plants. Further investigations will decipher mechanisms underlying the dynamics of community composition in both rhizosphere and root nodules and their involvement in other functions, which is important for the development of elite soybean varieties. 

## Figures and Tables

**Table 1:** Diversity indices of bacterial community in the rhizosphere soil of 4 varieties of soybean.

Variety	GS	Sobs	Coverage(%)	Chao	Ace	Shannon	Invsimpson	Npshannon
KA	BS	2110.0 ± 65.4a	0.9 ± 0.0	2875.1 ± 177.6ab	3037.2 ± 169.4a	6.1 ± 0.0ab	84.1 ± 4.3ab	6.3 ± 0.0a
	V2	2255.0 ± 55.5a	0.9 ± 0.0	3026.2 ± 223.6b	3181.8 ± 222.3a	6.3 ± 0.1b	114.8 ± 32.7bc	6.5 ± 0.1a
	R1	2230.7 ± 183.9a	1.0 ± 0.0	2826.2 ± 475.8ab	2962.2 ± 524.9a	6.4 ± 0.1b	140.4 ± 17.5bd	6.6 ± 0.1a
	R3	2004.7 ± 152.7ab	1.0 ± 0.0	2721.0 ± 333.3ab	3039.6 ± 539.2a	5.8 ± 0.4ab	59.4 ± 30.5ac	6.0 ± 0.4ab
	R5	2044.7 ± 117.9ab	1.0 ± 0.0	2621.8 ± 123.2ab	2755.7 ± 133.5a	6.1 ± 0.3ab	79.1 ± 46.1ab	6.2 ± 0.3ab
	R7	1612.3 ± 274.3b	1.0 ± 0.0	2185.0 ± 287.8a	2308.4 ± 261.1a	5.0 ± 0.9ac	30.2 ± 25.7a	5.1 ± 0.9b
PS	BS	2024.0 ± 128.3a	1.0 ± 0.0	2689.3 ± 239.2a	2815.0 ± 252.2a	6.0 ± 0.1a	73.5 ± 7.2a	6.2 ± 0.1a
	V2	2335.0 ± 183.4a	0.9 ± 0.0	3156.0 ± 546.0a	3512.8 ± 843.2a	6.4 ± 0.1a	110.2 ± 7.2a	6.5 ± 0.1a
	R1	2397.7 ± 191.6a	0.9 ± 0.0	3224.7 ± 480.2a	3387.5 ± 514.2a	6.5 ± 0.1a	147.2 ± 64.3a	6.7 ± 0.1a
	R3	2076.3 ± 113.9a	1.0 ± 0.0	2728.8 ± 151.7a	2852.6 ± 145.1a	6.0 ± 0.3a	75.7 ± 36.7a	6.2 ± 0.3a
	R5	2252.0 ± 108.1a	1.0 ± 0.0	2835.8 ± 200.2a	2979.6 ± 190.0a	6.4 ± 0.1a	127.1 ± 15.1a	6.5 ± 0.1a
	R7	2055.3 ± 257.5a	0.9 ± 0.0	2789.6 ± 398.7a	2942.4 ± 391.5a	6.1 ± 0.4a	105.6 ± 51.2a	6.3 ± 0.3a
PW	BS	2113.3 ± 63.3a	0.9 ± 0.0	2836.3 ± 88.0a	2983.7 ± 130.1a	6.2 ± 0.1ab	98.3 ± 23.4ab	6.3 ± 0.1ab
	V2	2182.3 ± 446.2a	0.9 ± 0.0	2952.6 ± 926.4a	3284.5 ± 1210.4a	6.2 ± 0.4ab	103.2 ± 43.2ab	6.4 ± 0.4b
	R1	2380.7 ± 309.2a	0.9 ± 0.0	3321.0 ± 697.2a	3474.1 ± 693.9a	6.4 ± 0.2a	149.5 ± 46.1a	6.6 ± 0.2b
	R3	2062.7 ± 68.5a	1.0 ± 0.0	2658.1 ± 5.0a	2783.4 ± 8.1a	6.1 ± 0.2ab	83.5 ± 26.8ab	6.2 ± 0.2ab
	R5	2066.7 ± 185.0a	1.0 ± 0.0	2661.0 ± 324.6a	2792.4 ± 256.3a	6.2 ± 0.1ab	108.1 ± 10.8ab	6.3 ± 0.1ab
	R7	1862.3 ± 101.7a	1.0 ± 0.0	2617.1 ± 82.4a	2881.8 ± 347.0a	5.4 ± 0.6bc	31.3 ± 23.6bc	5.5 ± 0.6ac
TK	BS	2063.3 ± 123.2ab	1.0 ± 0.0	2730.5 ± 346.2a	2844.0 ± 336.1a	6.1 ± 0.1ab	89.2 ± 13.1ab	6.3 ± 0.1a
	V2	2246.7 ± 115.9a	0.9 ± 0.0	3132.7 ± 432.3a	3464.0 ± 732.1a	6.3 ± 0.0b	102.7 ± 2.0b	6.4 ± 0.0a
	R1	2212.3 ± 186.2a	0.9 ± 0.0	3073.3 ± 513.0a	3375.9 ± 756.7a	6.3 ± 0.2b	104.6 ± 51.3b	6.4 ± 0.2a
	R3	2105.0 ± 52.8ab	1.0 ± 0.0	2784.4 ± 168.5a	2909.4 ± 174.7a	6.2 ± 0.1ab	106.2 ± 13.5b	6.4 ± 0.1a
	R5	2141.3 ± 98.2ab	1.0 ± 0.0	2714.2 ± 202.7a	2874.4 ± 203.6a	6.2 ± 0.1ab	100.9 ± 13.2b	6.4 ± 0.1a
	R7	1792.7 ± 189.5b	1.0 ± 0.0	2447.5 ± 270.0a	2590.1 ± 253.3a	5.3 ± 0.8ac	29.2 ± 20.3ac	5.4 ± 0.7b

GS, Growth stage of soybean (V2, R1, R3, R5, R7); BS, Bulk soil before planting 4 varieties of soybean; KA, Kwangan; PS, Poongsannamul; PW, Poongwon; TK, Taekwang. Values are Mean ± SD from three replications, the significant differences based on Tukey’s HDS test (*p* ≤ 0.05) were indicated with different letters.

**Table 2:** Diversity indices of bacterial community in the root nodule of 4 varieties of soybean.

Variety	GS	Sobs	Coverage (%)	Chao	Ace	Shannon	Invsimpson	Npshannon
KA	V2	23 ± 1.4a	1.0 ± 0.0	33 ± 3.6a	46.8 ± 15.1a	0.5 ± 0.1ab	1.4 ± 0.1ab	0.5 ± 0.1ab
	R1	22 ± 2.2a	1.0 ± 0.0	37.7 ± 3.8a	55.8 ± 8.0a	0.2 ± 0.1a	1.1 ± 0.1bc	0.2 ± 0.1b
	R3	67.7 ± 27.4b	1.0 ± 0.0	100.3 ± 30.9a	139.2 ± 32.5a	0.3 ± 0.1a	1.1 ± 0.1bc	0.3 ± 0.1b
	R5	38.0 ± 17.1ab	1.0 ± 0.0	60.8 ± 40.3a	88.0 ± 68.1a	0.2 ± 0.1a	1.0 ± 0.0c	0.2 ± 0.1b
	R7	71.3 ± 14.1bc	1.0 ± 0.0	94.8 ± 36.0a	108.6 ± 49.2a	0.8 ± 0.3b	1.5 ± 0.2a	0.9 ± 0.3ac
PS	V2	32 ± 9.2a	1.0 ± 0.0	60.3 ± 16.5a	73.9 ± 13.2a	0.2 ± 0.1a	1.1 ± 0.0a	0.2 ± 0.1a
	R1	31.3 ± 11.0a	1.0 ± 0.0	43.4 ± 9.6a	56.3 ± 4.2a	0.2 ± 0.1a	1.1 ± 0.1a	0.2 ± 0.1a
	R3	53.0 ± 6.2a	1.0 ± 0.0	82.0 ± 8.0a	85.1 ± 12.6a	0.2 ± 0.1a	1.1 ± 0.0a	0.2 ± 0.1a
	R5	28.0 ± 14.2a	1.0 ± 0.0	44.9 ± 22.2a	74.0 ± 34.6a	0.4 ± 0.1ab	1.2 ± 0.1a	0.4 ± 0.1ab
	R7	118.7 ± 75.0a	1.0 ± 0.0	138.5 ± 91.4a	148.7 ± 95.1a	1.1 ± 0.6b	1.6 ± 0.4a	1.1 ± 0.6bc
PW	V2	31.5 ± 0.5a	1.0 ± 0.0	46 ± 7.7a	65 ± 17.6a	0.2 ± 0.2a	1.1 ± 0.1a	0.2 ± 0.2a
	R1	25.7 ± 1.2a	1.0 ± 0.0	34.5 ± 6.7a	43.5 ± 10.6a	0.1 ± 0.1a	1.1 ± 0.1a	0.2 ± 0.1a
	R3	41.3 ± 8.7a	1.0 ± 0.0	74.1 ± 31.9ab	83.9 ± 31.5a	0.1 ± 0.1a	1.0 ± 0.0a	0.1 ± 0.1a
	R5	21.0 ± 8.6a	1.0 ± 0.0	24.3 ± 9.7a	34.8 ± 14.8a	0.1 ± 0.1a	1.1 ± 0.1a	0.1 ± 0.1a
	R7	83.0 ± 25.2b	1.0 ± 0.0	121.3 ± 37.0b	156.0 ± 31.2b	0.6 ± 0.5a	1.3 ± 0.3a	0.6 ± 0.5a
TK	V2	23.7 ± 0.9a	10. ± 0.0	48.4 ± 12.6ab	84.8 ± 45.8a	0.3 ± 0.2ab	1.2 ± 0.1a	0.3 ± 0.2ab
	R1	22.3 ± 3.9a	1.0 ± 0.0	36.0 ± 8.3a	46.1 ± 17.9a	0.1 ± 0.1a	1.1 ± 0.0a	0.1 ± 0.1a
	R3	47.3 ± 24.0a	1.0 ± 0.0	70.9 ± 23.0ab	92.2 ± 32.7a	0.3 ± 0.3ab	1.2 ± 0.2a	0.3 ± 0.3ab
	R5	29.3 ± 18.2a	1.0 ± 0.0	37.7 ± 24.3a	40.2 ± 24.9a	0.1 ± 0.1a	1.0 ± 0.0a	0.1 ± 0.1a
	R7	103.3 ± 61.4a	1.0 ± 0.0	133.0 ± 64.2b	132.5 ± 68.2a	1.2 ± 0.8bc	2.0 ± 1.0a	1.2 ± 0.8bc

GS, Growth stage of soybean (V2, R1, R3, R5, R7); KA, Kwangan; PS, Poongsannamul; PW, Poongwon; TK, Taekwang. Values are Mean ± SD from three replications, the significant differences based on Tukey’s HDS test (*p* ≤ 0.05) were indicated with different letters.

## Data Availability

Not applicable.

## Supplementary Materials

The following are available online at https://www.mdpi.com/article/10.3390/ijms22115577/s1; Figure S1: Bacterial diversity indices of bulk soil and rhizosphere of four soybean varieties. Figure S2: Bacterial diversity indices of root nodules of four soybean varieties. Figure S3: Influence of varieties and growth stages in the dominant phylum present in bulk soil and rhizosphere of soybean. Figure S4: Influence of varieties and growth stages in the dominant phylum present in root nodules of soybean. Figure S5: Influence of varieties and growth stages in the dominant genera present in bulk soil and rhizosphere of soybean. Figure S6: Influence of varieties and growth stages in the dominant genera present in root nodules of soybean. Table S1. Substrate utilization indicated by color development in Biolog Eco Plates. Table S2. Overall OTUs identified from the sequencing reads.
