# Supplementary material for: Dynamics of Bacterial Community Structure in the Rhizosphere and Root Nodule of Soybean: Impacts of Growth Stages and Varieties
Source: Int J Mol Sci. 2021 May 25;22(11):5577. doi: 10.3390/ijms22115577 (PMC8197538; doi:10.3390/ijms22115577)
Supplement: Supplementary file 1 [file ijms-22-05577-s001.zip › 4 Supplementary Figures.pdf]

Figure S1: Bacterial diversity indices of bulk soil and rhizosphere of four soybean varieties

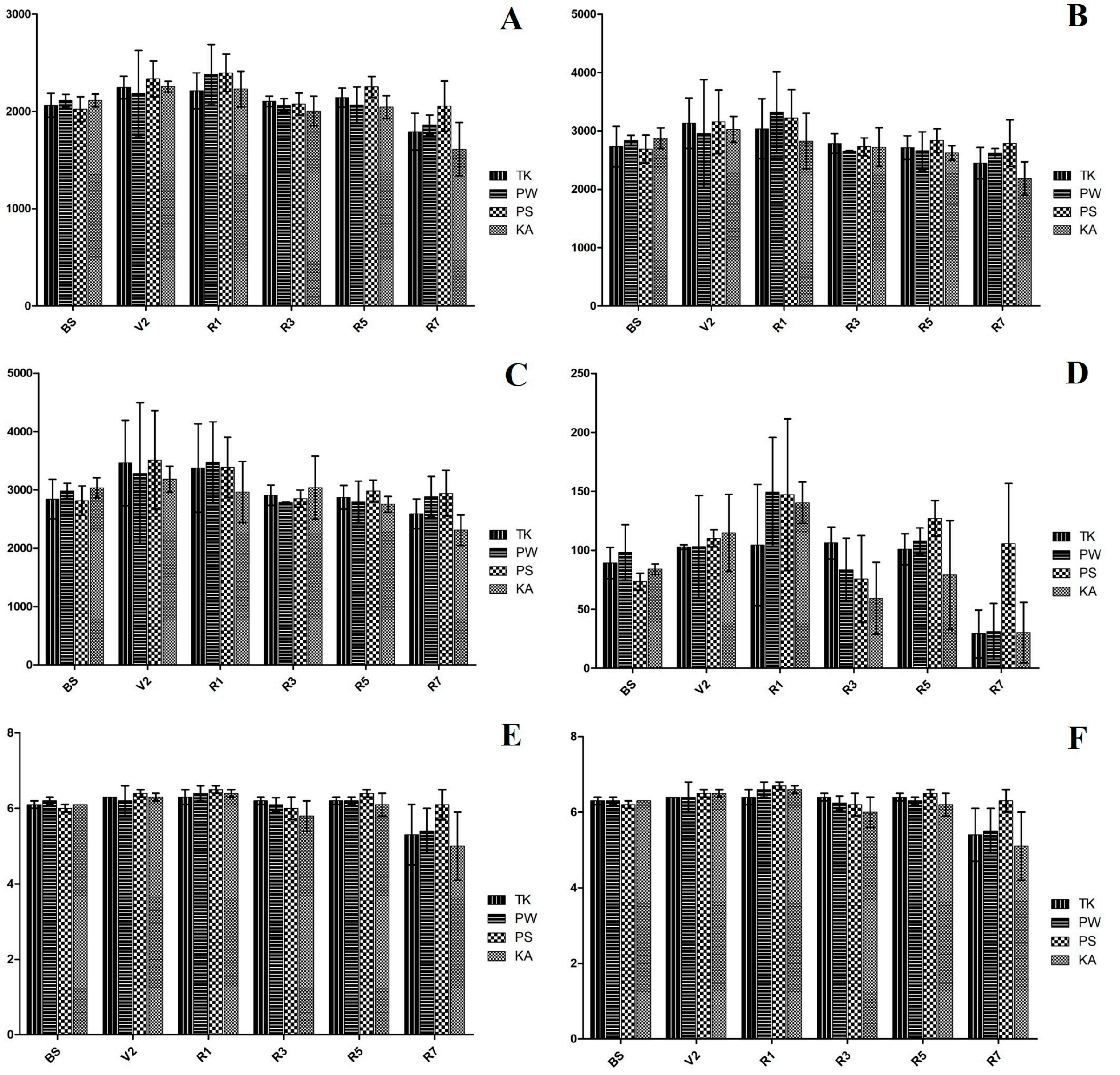

A, Sobs; B, Chao; C, Ace; D, Shannon; E, Invsimpson; F, Npshannon. V2, R1, R3, R5, R7, Growth stage of soybean; BS, Bulk soil before planting 4 varieties of soybean; KA, Kwangan; PS, Poongsannamul; PW, Poongwon; TK, Taekwang.

Figure S2: Bacterial diversity indices of root nodules of four soybean varieties

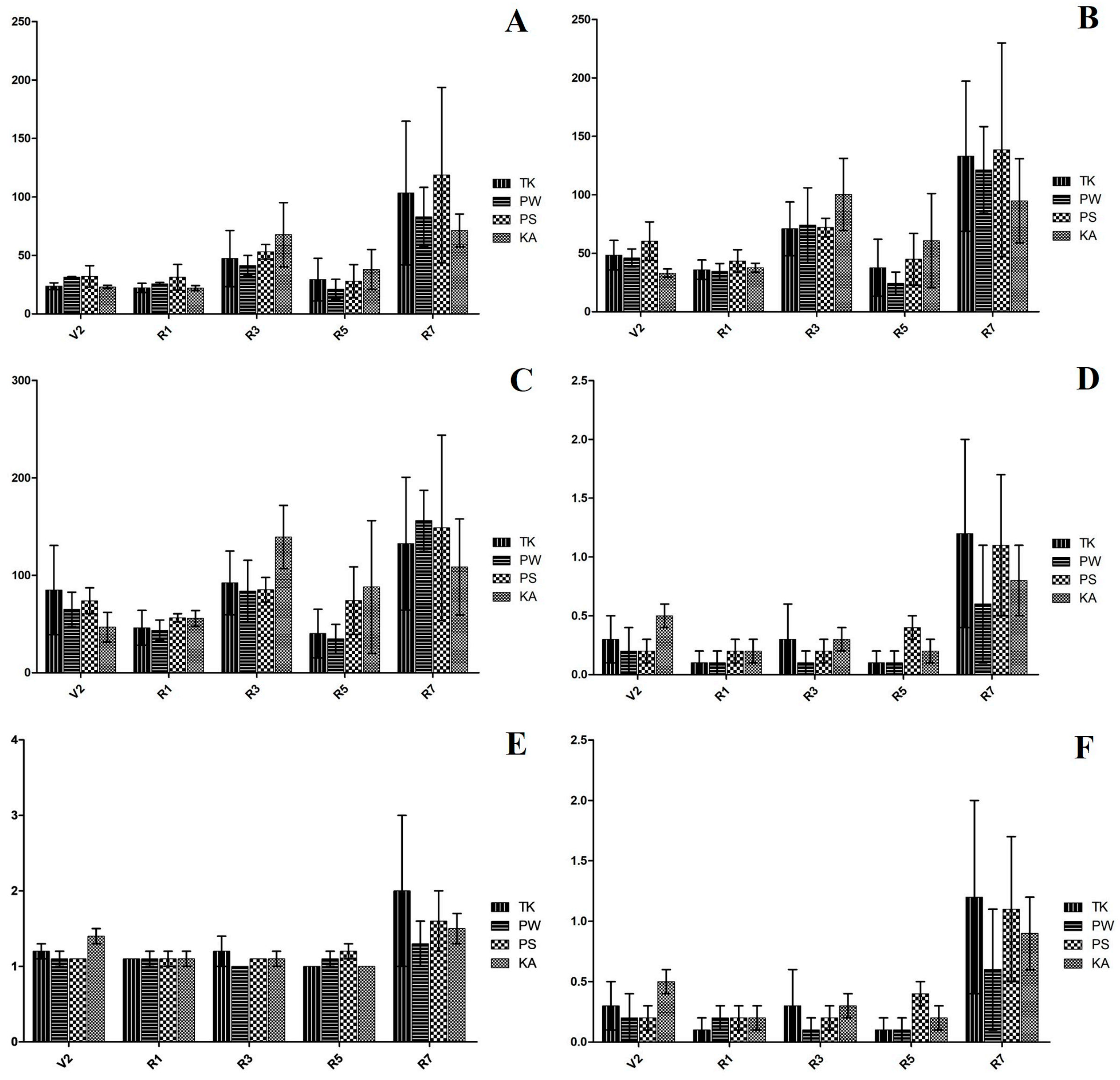

A, Sobs; B, Chao; C, Ace; D, Shannon; E, Invsimpson; F, Npshannon. V2, R1, R3, R5, R7, Growth stage of soybean; BS, Bulk soil before planting 4 varieties of soybean; KA, Kwangan; PS, Poongsannamul; PW, Poongwon; TK, Taekwang.

Figure S3: Influence of varieties and growth stages in the dominant phylum present in bulk soil and rhizosphere of soybean

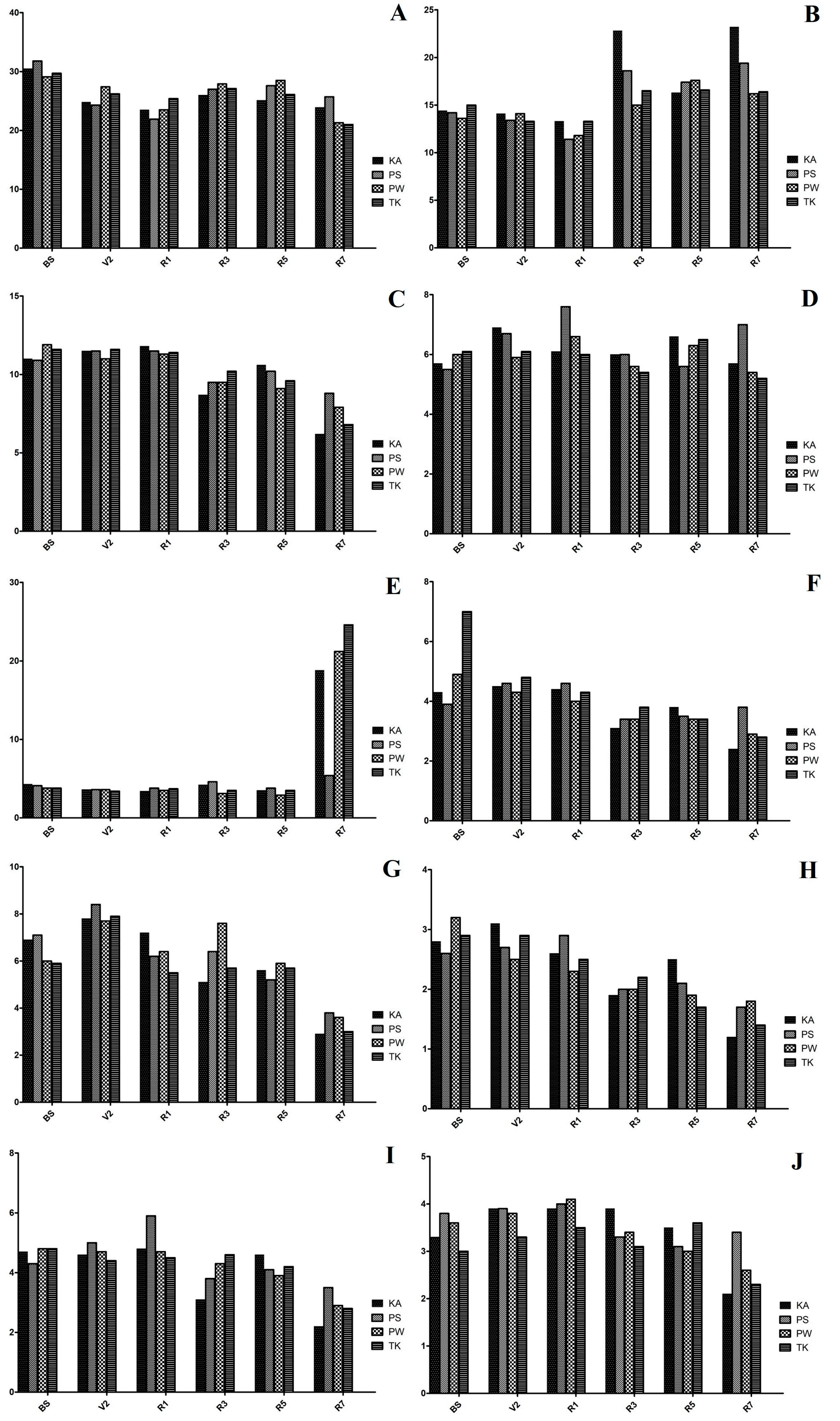

A, *Actinobacteria*; B, *Alphaproteobacteria*; C, *Acidobacteria*; D, *Betaproteobacteria*; E, *Bacteroidetes*; F, *Chloroflexi*; G, *Firmicutes*; H, *Gemmatimonadetes*; I, *Deltaproteobacteria*; J, *Gammaproteobacteria*. V2, R1, R3, R5, R7, Growth stages of soybean; BS, Bulk soil before planting, KA, Kwangan; PS, Poongsannamul; PW, Poongwon; TK, Taekwang (4 varieties of soybean).

Figure S4: Influence of varieties and growth stages in the dominant phylum present in root nodules of soybean

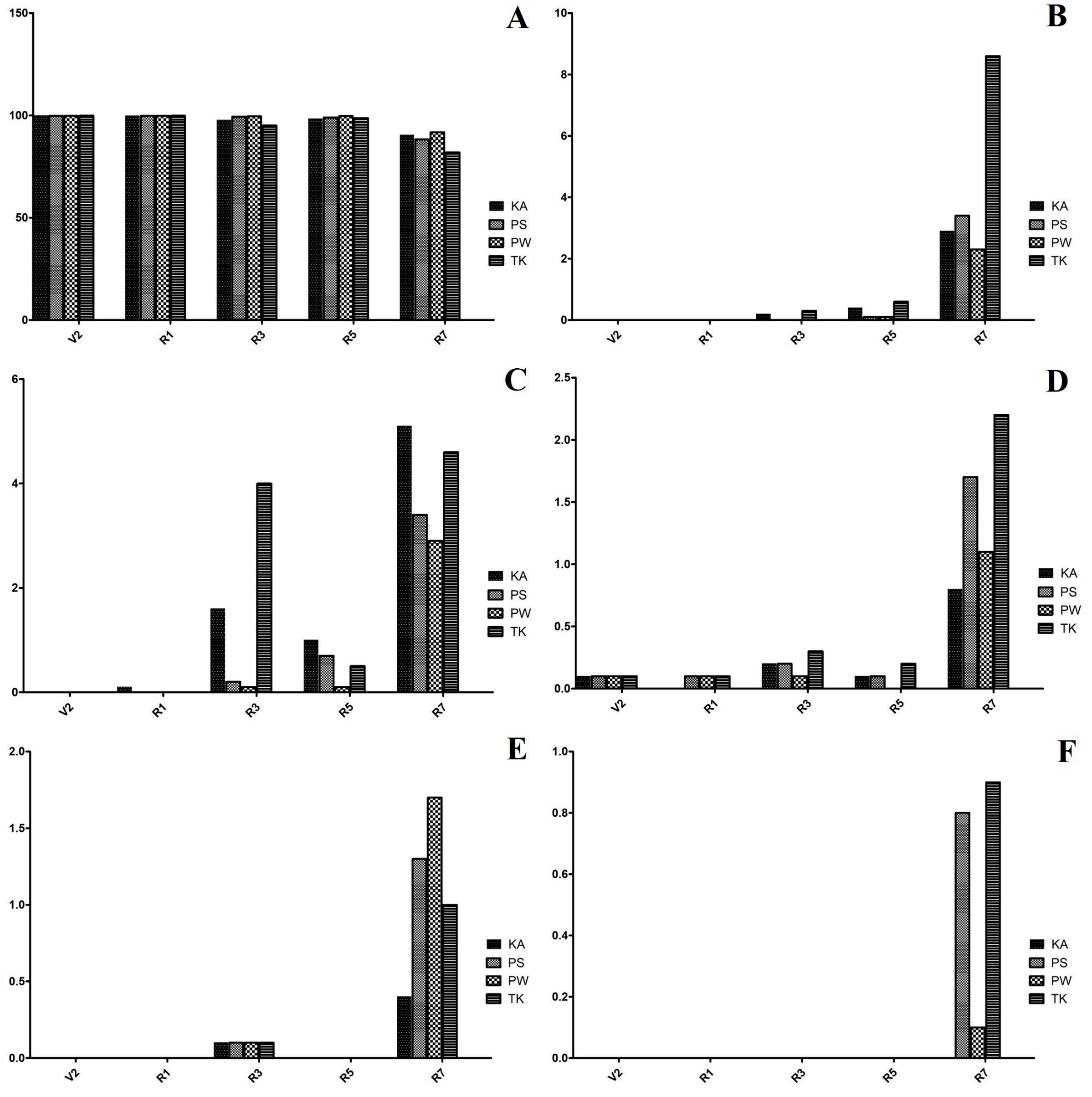

A, *Alphaproteobacteria*; B, *Bacteroidetes*; C, *Gammaproteobacteria*; D, *Betaproteobacteria*; E, *Actinobacteria*; F, *Verrucomicrobia*.V2, R1, R3, R5, R7, Growth stages of soybean; KA, Kwangan; PS, Poongsannamul; PW, Poongwon; TK, Taekwang (4 varieties of soybean).

Figure S5: Influence of varieties and growth stages in the dominant genera present in bulk soil and rhizosphere of soybean

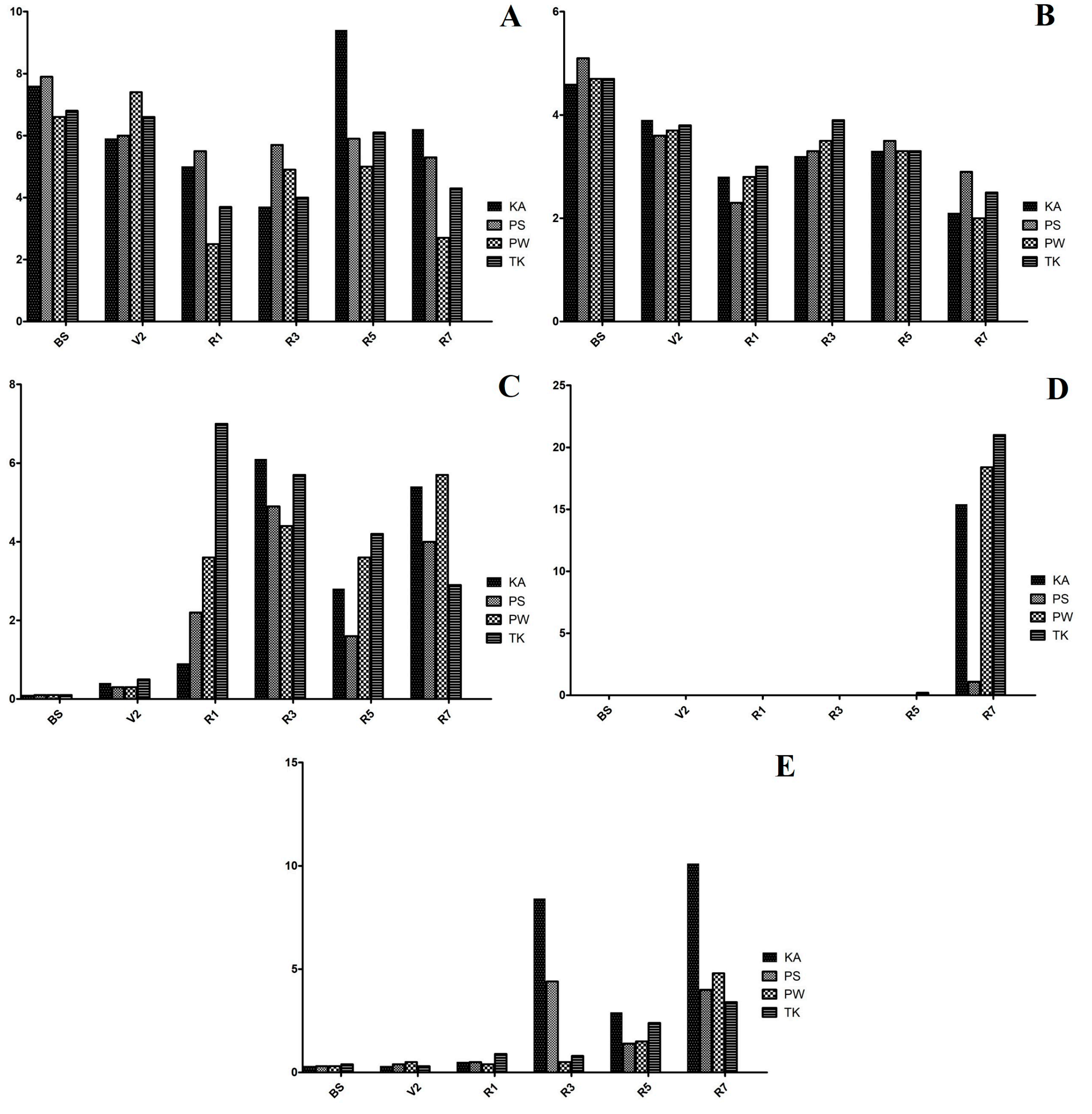

A, *Arthrobacter*; B, *Sphingomonas*; C, *Aeromicrobium*; D, *Flavobacterium*; E, *Bradyrhizobium*.V2, R1, R3, R5, R7, Growth stages of soybean; BS, Bulk soil before planting, KA, Kwangan; PS, Poongsannamul; PW, Poongwon; TK, Taekwang (4 varieties of soybean).

Figure S6: Influence of varieties and growth stages in the dominant genera present in root nodules of soybean

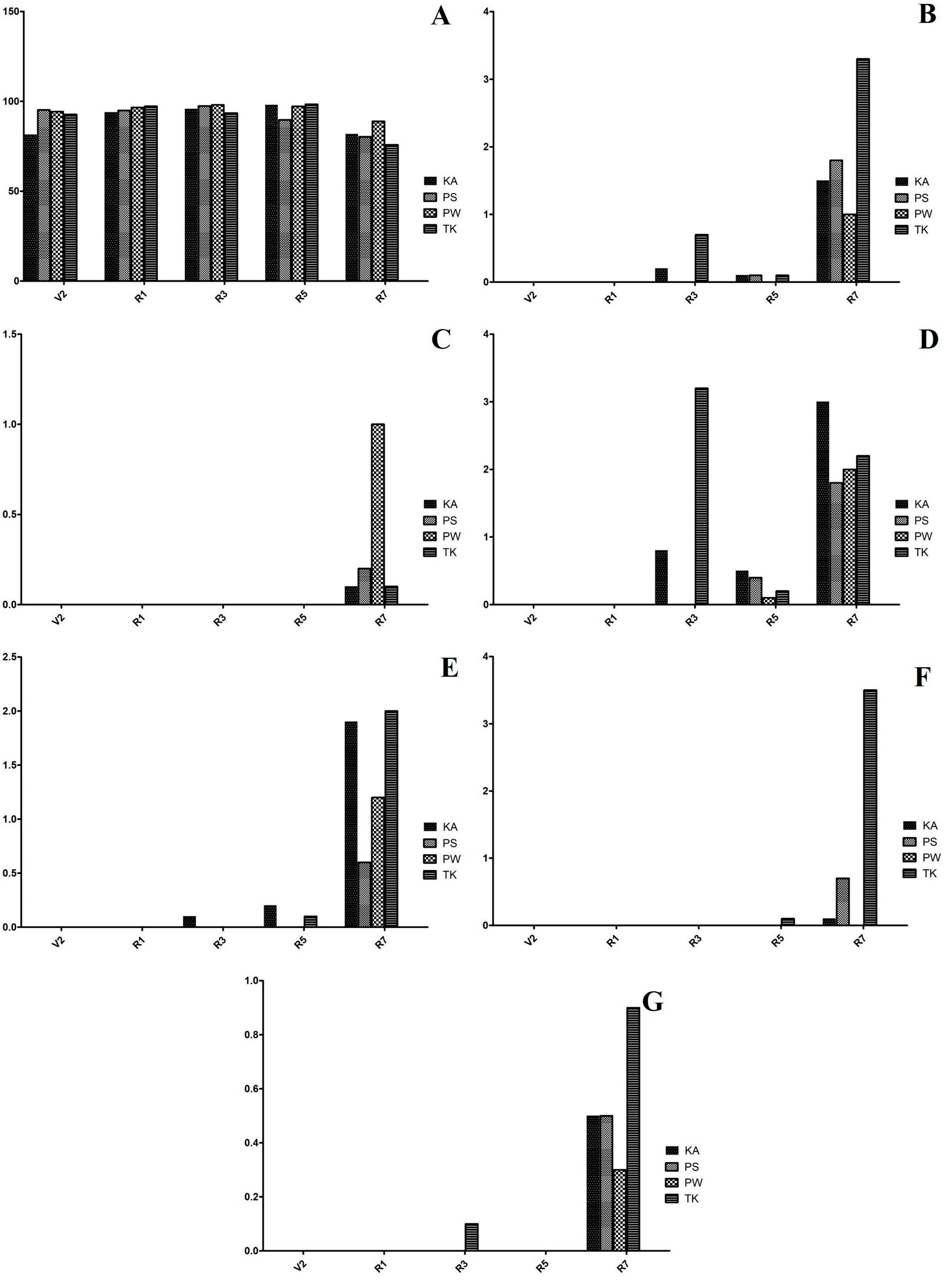

A, *Bradyrhizobium*; B, *Rhizobium*; C, *Streptomyces*; D, *Enterobacter*; E, *Sphingobacterium*; F, *Flavobacterium*; G, *Achromobacter*.V2, R1, R3, R5, R7, Growth stages of soybean; KA, Kwangan; PS, Poongsannamul; PW, Poongwon; TK, Taekwang (4 varieties of soybean).
